# The Protective Effects of Sulforaphane on High-Fat Diet-Induced Obesity in Mice Through Browning of White Fat

**DOI:** 10.3389/fphar.2021.665894

**Published:** 2021-04-29

**Authors:** Yaoli Liu, Xiazhou Fu, Zhiyong Chen, Tingting Luo, Chunxia Zhu, Yaoting Ji, Zhuan Bian

**Affiliations:** ^1^ Key Laboratory Breeding Base of Basic Science of Stomatology (Hubei-MOST) and Key Laboratory for Oral Biomedicine of Ministry of Education, School and Hospital of Stomatology, Wuhan University, Wuhan, China; ^2^ Center of Stomatology, Tongji Hospital of Tongji Medical College of Huazhong University of Science and Technology, Wuhan, China

**Keywords:** sulforaphane, obesity, white adipose browning, browning, high-fat diet

## Abstract

**Background:** Sulforaphane (SFN), an isothiocyanate naturally occurring in cruciferous vegetables, is a potent indirect antioxidant and a promising agent for the control of metabolic disorder disease. The glucose intolerance and adipogenesis induced by diet in rats was inhibited by SFN. Strategies aimed at induction of brown adipose tissue (BAT) could be a potentially useful way to against obesity. However, *in vivo* protective effect of SFN against obesity by browning white adipocyte has not been reported. Our present study is aimed at evaluation the efficacy of the SFN against the high-fat induced-obesity mice and investigating the potential mechanism.

**Methods:** High-Fat Diet-induced obese female C57BL/6 mice were intraperitoneally injected with SFN (10 mg/kg) daily. Body weight was recorded every 3 days. 30 days later, glucose tolerance test (GTT) and insulin tolerance test (ITT) were performed. At the end of experiment, fat mass were measured and the adipogenesis as well as browning associated genes expression in white adipose tissue (WAT) were determined by RT-qPCR and western blot. Histological examination of the adipose tissue samples were carried out with hematoxylin–eosin (HE) staining and immunofluorescence staining method. *In vitro*, pre-adipocytes C3H10T1/2 were treated with SFN to investigate the direct effects on adipogenesis.

**Results:** SFN suppressed HFD-induced body weight gain and reduced the size of fat cells in mice. SFN suppressed the expression of key genes in adipogenesis, inhibited lipid accumulation in C3H10T1/2 cells, increased the expression of brown adipocyte-specific markers and mitochondrial biogenesis *in vivo* and *in vitro*, and decreased cellular and mitochondrial oxidative stress. These results suggested that SFN, as a nutritional factor, has great potential role in the battle against obesity by inducing the browning of white fat.

**Conclusion:** SFN could significantly decrease the fat mass, and improve glucose metabolism and increase insulin sensitivity of HFD-induced obese mice by promoting the browning of white fat and enhancing the mitochondrial biogenesis in WAT. Our study proves that SFN could serve as a potential medicine in anti-obesity and related diseases.

## Introduction

Obesity is a global public health problem that results in many metabolic disorders, such as type 2 diabetes, coronary heart disease, cancer, hypertension, and other chronic diseases (Kopelman, 2007). These diseases have an adverse effect on personal health and cause the cost explosion in public health systems (Ahima and Lazar, 2013).

There are two main types of adipose tissue in mammals: white adipose tissue (WAT) and brown adipose tissue (BAT) (Jeremic et al., 2017). WAT, which is composed of subcutaneous and visceral fat, is specialized to store energy and directly modulates metabolism by secreting various adipokines, such as LPL and LEP (Plaza et al., 2018). In comparison, BAT is highly expressing uncoupling protein 1 (UCP1) and contains a considerable amount of mitochondria, which enhances heat production and thermogenesis (Bargut et al., 2017).

Although white and brown adipocytes originate from different cell lineages, they are readily interconvertible to each other (Sanchez-Gurmaches et al., 2016). The *Ucp1* positive multilocular cells with thermogenic capability developed in white fat depots, which are characterized as beige or brite (brown in white), with some external stimuli (Herz and Kiefer, 2019). WAT can possess biochemical and morphological features similar to those of BAT, such as the presence of multilocular lipid droplets and the increased expression of *Ucp1* and other genes associated with mitochondrial biogenesis, when subjected to certain stimuli (Vidal and Stanford, 2020). The conversion process of WAT into BAT is called “browning”, which may positively contribute to cellular bioenergetics and metabolism homeostasis (Herz and Kiefer, 2019). WAT could be appearance of brite adipocytes under the condition of cold exposure or pharmacological activaton β-3 adrenergic receptors *via* the promotion of WAT reprogramming (P. Liu et al., 2019b).

It was well known that a number of dietary compounds present in fruit and vegetables could increase energy expenditure and decrease fat accumulation in mammals resulting in the decrease of medical care cost associated with obesity (Montanari et al., 2017). Sulforaphane (SFN), derived from glucosinolates in cruciferous vegetables, is a potent indirect anti-oxidant and as a promising chemopreventive agent against cancer (Houghton, 2019). More and more research proved that SFN may play a positive role in the target to control metabolic disorder (Z. Li et al., 2020b). The glucose intolerance and adipogenesis induced by diet in rats was inhibited by SFN (Sun et al., 2020). SFN induces the browning of pre-adipocytes via increasing the content of mitochondrial and activity of enzymes in respiratory chain (Zhang et al., 2016). However, whether SFN prevent high fat feed-induced obesity through the browning of white adipocyte remains unknown.

In the present study, we report that SFN induces the browning of mature C3H10T1/2 adipocytes based on the promotion of mitochondrial biogenesis by means of the upregulation of the AMPK and NRF2 signaling pathways and the enhancement of mitochondrial function. Our further research revealed that SFN can prevent high-fat diet (HFD)-induced obesity in C57BL/6N mice by inducing the browning of WAT.

## Materials and Methods

### Cell Culture and Differentiation

C3H10T1/2 pre-adipocytes were cultured in basal medium (high-glucose Dulbecco’s modified Eagle’s medium [DMEM]) supplemented with 10% FBS, 1% penicillin-streptomycin, and 1% L-glutamine) at 37°C in a humidified incubator with 5% CO_2_. Cells were passaged when 80% confluent, then seeded in a six-well plate at a density of 1 × 10^5^ cells/well, and grown to confluency (designated as “day 0”) in basal medium. The differentiation of the cells into mature adipocytes was induced through the classic cocktail method. At day 0, the cells were cultured in DMEM with 10% FBS, 0.5 μM rozglitazone, 10 μM insulin, 0.25 μM glutamine, 1 μM dexamethasone, and 0.5 mM 3-isobutyl-1-methyxanthine for 3 days. The culture medium was replaced with DMEM containing 10% FBS, 0.25 μM glutamine, and 10 μM insulin at day 3 for 1 day. The steps were repeated three times until the cells differentiated into mature adipocytes.

### Cell Viability Assay

C3H10T1/2 cells were seeded at a density of 3,000 cells/well in a 96-well plate. After 24 h, the cells were treated with various concentrations of SFN (0, 1, 5, 10, and 20 μM) (Cayman Chemical, Item No: 10,496, Purity ≥98%, synthetic, dissolved in PBS). After 12, 24, or 48 h of culture, the reagent of cell counting kit 8 (CCK8, 10 μL) was added to each well. The plates were incubated for another 1 h at 37°C. The absorbance was measured at 450 nm.

### Oil Red O Staining

Oil red O staining was performed after the C3H10T1/2 cells differentiated into mature adipocytes. The cells were washed with PBS and fixed with 4% formalin for 30 min. The lipid droplets were stained with Oil Red O solution for 20 min. The solution was removed, and the lipid droplets in the cells were dissolved in 100% isopropanol. Optical density was measured at 520 nm.

### Real-Time Quantitative Polymerase Chain Reaction Analysis

Total RNA was isolated using the TRIzol^®^ (TaKaRa Bio, Otsu, Shiga, Japan) method following the manufacturer’s instruction. The concentration and quality of the total RNA was checked by NanoDrop 2000 analyzer (Thermo Scientific, Ltd., Wilmington, DE, United States). cDNA was synthesized using the HiScript II QRT SuperMix for qPCR (+gDNA wiper) (Vazyme biotech). qPCR was performed with the QuantStudio six Flex Real-Time PCR System (Life Technologies, United States) using ChamQ SYBR qPCR Master Mix (Vazyme biotech). All reactions were performed in triplicate. Fold differences in gene expression were calculated with the ΔΔCt method using β-actin as the housekeeping gene. The primer sequences for tested genes were as follows (Table 1).

**TABLE 1:** Oligonucleotide Primers Used for qPCR.

Gene name	Forward primer (5–3′)	Reverse primer (5–3′)
*Ppar γ*	GGA​AGA​CCA​CTC​GCA​TTC​CTT	GTA​ATC​AGC​AAC​CAT​TGG​GTC​A
*Fas*	AAC​TCA​CTG​GCA​GAA​GAG​AA	CTT​CAA​GAA​GAT​AGC​CAT​GC
*Cebpβ*	GCCCGTTGCCAGGCG	TGG​CCA​CTT​CCA​TGG​GTC​TA
*Cebp*α	CAA​GAA​CAG​CAA​CGA​GTA​CCG	GTC​ACT​GGT​CAA​CTC​CAG​CAC
*Scd1*	ATG​GAT​ATC​GCC​CCT​ACG​AC	GAT​GTG​CCA​GCG​GTA​CTC​AC
*Chop*	GCA​GCG​ACA​GAG​CCA​GAA​TA	ATG​TGC​GTG​TGA​CCT​CTG​TT
*Tmem26*	GAA​ACC​AGT​ATT​GCA​GCA​CCC	CCA​GAC​CGG​TTC​ACA​TAC​CA
*Ucp1*	CCT​GCC​TCT​CTC​GGA​AAC​AA	GTA​GCG​GGG​TTT​GAT​CCC​AT
*Hsl*	TAT​GGA​GTG​AGG​GTG​CCA​GA	ATG​GTC​CTC​TGC​CTC​TGT​CC
*Cpt1*α	GTG​TTG​GAG​GTG​ACA​GAC​TT	CAC​TTT​CTC​TTT​CCA​CAA​GG
*Prdm16*	GAT​GGG​AGA​TGC​TGA​CGG​AT	TGA​TCT​GAC​ACA​TGG​CGA​GG
*β-actin*	CGT​GCG​TGA​CAT​CAA​AGA​GAA	GCT​CGT​TGC​CAA​TAG​TGA​TGA
*COX 1*	ACT​ATA​CTA​CTA​CTA​ACA​GAC​CG	GGT​TCT​TTT​TTT​CCG​GAG​TA
*cyclophilin A*	ACA​CGC​CAT​AAT​GGC​ACT​GG	CAG​TCT​TGG​CAG​TGC​AGA​T

### Animal Models

The animal procedures were approved by the Institutional Animal Care and Use Committee of Wuhan University. Female C57BL/6 mice (*n* = 28, 8 weeks old) were purchased from the Laboratory Animal Center of Wuhan University (Wuhan, China) and maintained at a temperature of 24–26°Cand a 12 h light/12 h dark cycle. The mice were randomly divided into four groups: group 1, normal diet; group 2, normal diet plus SFN (10 mg/kg/day); group 3, HFD; and group 4, HFD plus SFN (10 mg/kg/day). In group 2 and 4, C57BL/6 mice were intraperitoneally injected with SFN (10 mg/kg) daily and in group 1 and 3, mice were intraperitoneally injected with the same amount of PBS.

### Glucose Tolerance Test and Insulin Tolerance Test

After 30 days intervention, the GTT and ITT were performed. For GTT, the mice were fasted for 16 h of the next day with free access to drinking water. The blood glucose were recorded before the mice were intraperitoneally injected with 10% D-glucose (1.0 g/kg body weight). Blood glucose levels were measured at 15, 30, 60, 90, and 120 min with Sannuo blood glucose meter (Sinocare Inc. China). Three days later, the mice were subjected to ITT. The mice were fasted for 4 h (8:00–12:00) with free access to drinking water and then intraperitoneally injected with insulin (1 U/kg body weight; Humulin; Eli Lilly, Indianapolis, IN, United States ). Blood glucose levels were measured as described above. The animals were euthanized with Carbon dioxide (CO_2_) inhalation 3 days after the ITT was performed and tissue collection was performed at this stage.

### Histological and Immunostaining Analyses

Adipose and liver tissues were fixed in 4% paraformaldehyde solution, dehydrated in a graded ethanol series (70–100%), and embedded in paraffin. Paraffin-embedded section was sliced at 5 µm (*Leica*, RM2235). The slides were carried out with hematoxylin–eosin (HE) staining and subjected to immunohistochemistry staining with UCP-1 antibody (Ab10983; Abcam). The image were captured with a light microscope (CX41; Olympus, Japan) and analyzed using NIH ImageJ software.

### Western Blot

C3H10T1/2 cells were washed with ice-cold PBS for three times and lyzed with lysis buffer at 4°C for 30 min and further treated by ultrasound, then centrifuged at 12,000 rpm for 15 min at 4°C. The supernatant was collected and the protein concentrations were determined by BCA™ protein assay. Samples were loaded and separated in 12% sodium dodecyl sulfate polyacrylamide (SDS-PAGE) gels and then transferred to polyvinylidene difluoride (PVDF) membrane. The membrane was blocked in 5% non-fat milk for 15 min and incubated overnight at 4°C with primary antibodies. The details of the primary antibodies used are as followed: Akt (4,685; Cell Signaling Technology), phos-Akt (4,060; Cell Signaling Technology), p38 (8,690; Cell Signaling Technology), phos-p38 (4,511; Cell Signaling Technology), JNK (9,252; Cell Signaling Technology), phos-JNK (4,668; Cell Signaling Technology), Erk (4,695; Cell Signaling Technology), phos-Erk (4,370; Cell Signaling Technology), CREB (9,197; Cell Signaling Technology), phos-CREB (9,198; Cell Signaling Technology), UCP-1 (Ab10983; Abcam),PGC1α (66369-1-Ig; proteintech), NRF2 (16396-1-AP; proteintech), GAPDH (10494-1-AP; proteintech), ACTB (20536-1-AP; proteintech). All the primary antibodies were incubated at the concentration of 1:1,000. Then, the membrane was washed with Tris-buffered saline with Tween and incubated with secondary antibodies at 25°C for 1 h. The images were presented using ECL luminous fluid (Advansta, America) on the imaging system (Li-cor Odyssey, America).

### MitoSceneTM Green II Staining

Removed the medium from the dishes, and the cells were washed with ice-cold PBS. The prewarmed solution containing 100 nM MitoScene TM Green II (US EVERBRIGHT, Jiangsu, China) was added and incubated in the dark for 30 min at 37°C. Then the cells were observed using a fluorescence microscope. The paraffin sections of adipose tissues were hydrated in a graded ethanol series (70–100%). MitoScene TM Green II (US EVERBRIGHT, Jiangsu, China) was used to determine the mitochondrial mass. The tissues were stained with mitochondria-specific dye at 37°C for 30 min and 4′6-diamidino-2-phenylindole for 15 min. Digital images (×200) were captured with a fluorescent microscope.

### Measurement of Mitochondrial DNA Copy Number

QIA amp DNA Mini Kit (Qiagen, Venlo, Netherlands) was used to extract total DNA (genomic and mitochondrial DNA) in C3H10T1/2 cells following the instructions. The concentration and quality of DNA were determined using NanoDrop 2000 analyzer (Thermo Scientific, United States). qPCR was performed with the QuantStudio six Flex Real-Time PCR System (Life Technologies, United States) using ChamQ SYBR qPCR Master Mix (Vazyme biotech) to analyze the copy number of mtDNA associated with genomic DNA. The primer sequences of COX one and cyclophilin A are shown in Table 1.

### Adipocyte Size Analysis

The section images of HE staining were used to calculate the individual adipocyte size (square area) by the delineation of the circumference of individual adipocytes using the NIH ImageJ software. For each image, the area of the individual adipocytes were sorted from the smallest to the largest. Subsequently, the total number of adipocytes and the mean adipocyte area were determined.

### Statistical Analyses

All data are presented as mean ± standard deviation (SD). Statistical analysis was performed using SPSS software. One-way ANOVA and Student t test was used to analysis the difference. *p* < 0.05 was considered statistically significant.

## Results

### Sulforaphane Inhibits the Adipogenesis and Induces the Browning of C3H10T1/2 Cells

**FIGURE 1:**
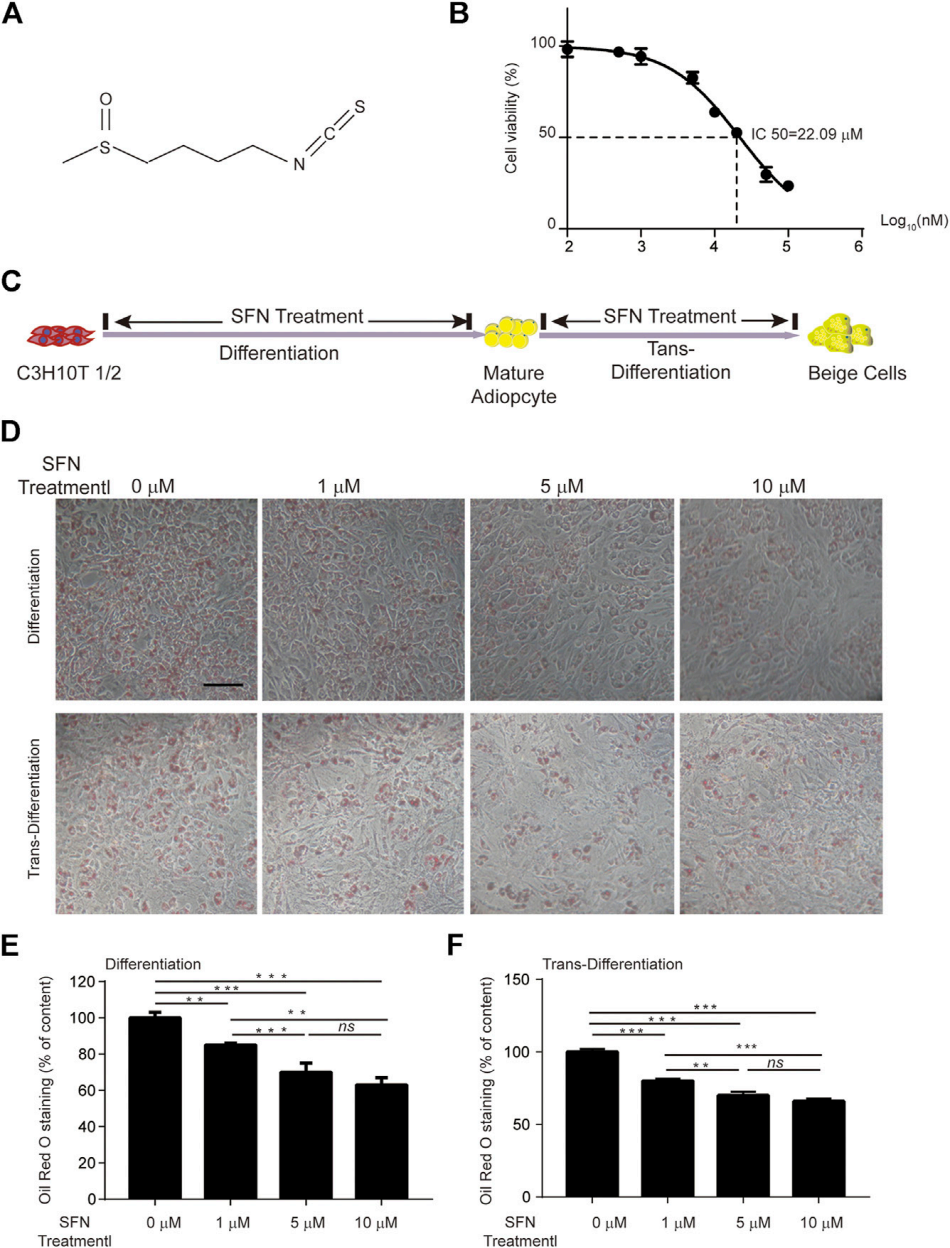
Effects of SFN on lipid droplet biogenesis during the differentiation o*r* trans-differentiation of C3H10T1/2 cells. **(A)** Chemical structure of SFN. **(B)** IC50 of SFN on C3H10T1/2 cells. **(C)** Flow diagram of the experimental design **(D)** SFN inhibited the lipid accumulation in the differentiation of C3H10T1/2 cells and enhanced the trans-differentiation of adipocytes. Cells were fixed and stained with Oil red O. **(E,F)** Oil red O staining was quantified. Scar bar = 50 μm. All the results were expressed in graph with mean ± SD. Statistical significance was evaluated by ANOVA test. *, ** and *** represent the significant difference at *p* < 0.05, *p* < 0.001 and *p* < 0.0001. *ns* represent not significant.

SFN is a small molecular compound with high energy chemical bonds (Figure 1A). The cell viability of C3H10T1/2 cells treated with different SFN concentrations was detected using CCK8 assays. The results revealed that the half-maximal inhibitory concentration (IC50) of SFN to C3H10T1/2 cells is 20 μM (Figure 1B). Thus, the maximum concentration we used *in vitro* experiment was 10 μM.

**FIGURE 2:**
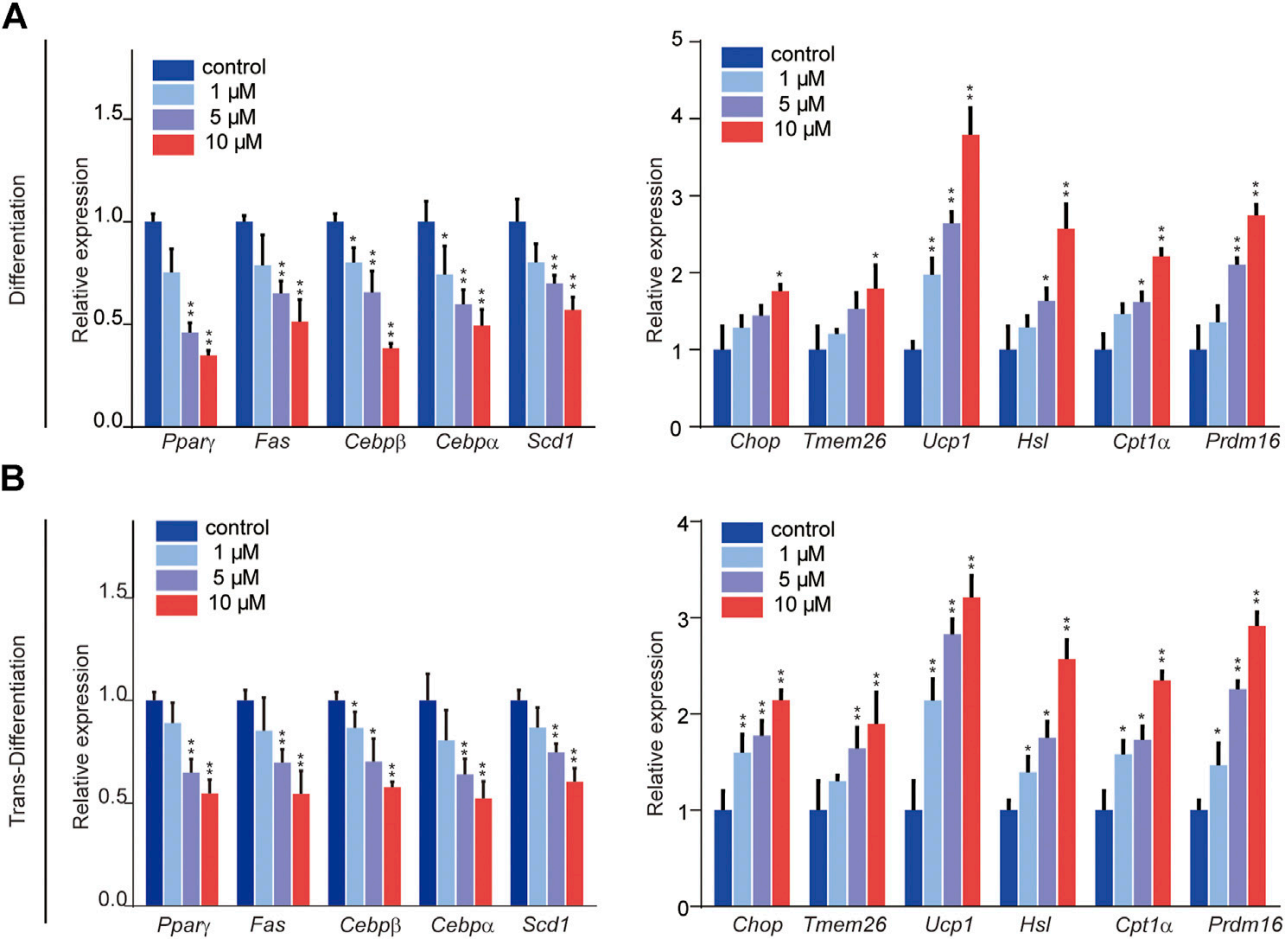
Effects of SFN on the expression of key adipocyte-specific markers **(left)** and browning-specific markers **(right)** in C3H10T1/2 adipocytes. **(A,B)** Total RNA was extracted at the end of the differentiation and trans-differentiation processes. mRNA expression was analyzed by real-time qPCR. The results were normalized to β-actin. All the results were expressed in graph with mean ± SD. Statistical significance was evaluated by Student t test. * and ** represent the significant difference at *p* < 0.05, *p* < 0.01 vs control group.

The processes of adipogenic differentiation and browning were designed as shown in Figure 1C. During the induction of adipogenic differentiation, the cells were incubated with varying SFN concentrations (1–10 μM). We found that SFN considerably inhibits the differentiation of C3H10T1/2 cells into mature adipocytes and lipid content (Figures 1D,E). Furthermore, the lipid droplet decreased when the mature adipocytes were treated with SFN (Figures 1D,F). Mature adipocytes could trans-differentiate into other cell types, like beige cells, under certain conditions. The gene expression profile of C3H10T1/2 cells after SFN treatment showed that SFN inhibited the expression of core adipogenesis genes (*Ppar*γ*, Fas, Cebp*β and *Scd1*) and enhanced the expression of browning genes (*Chop, Temem 26, Ucp1, Pgc-1*α*,* and *Prdm16*) in adipocyte differentiation and trans-differentiation. This result suggested the possible conversion of white adipocytes into beige cells (Figures 2A,B).

### SFN Attenuates Weight Gain and Adipogenesis Through the Browning of WAT

**FIGURE 3:**
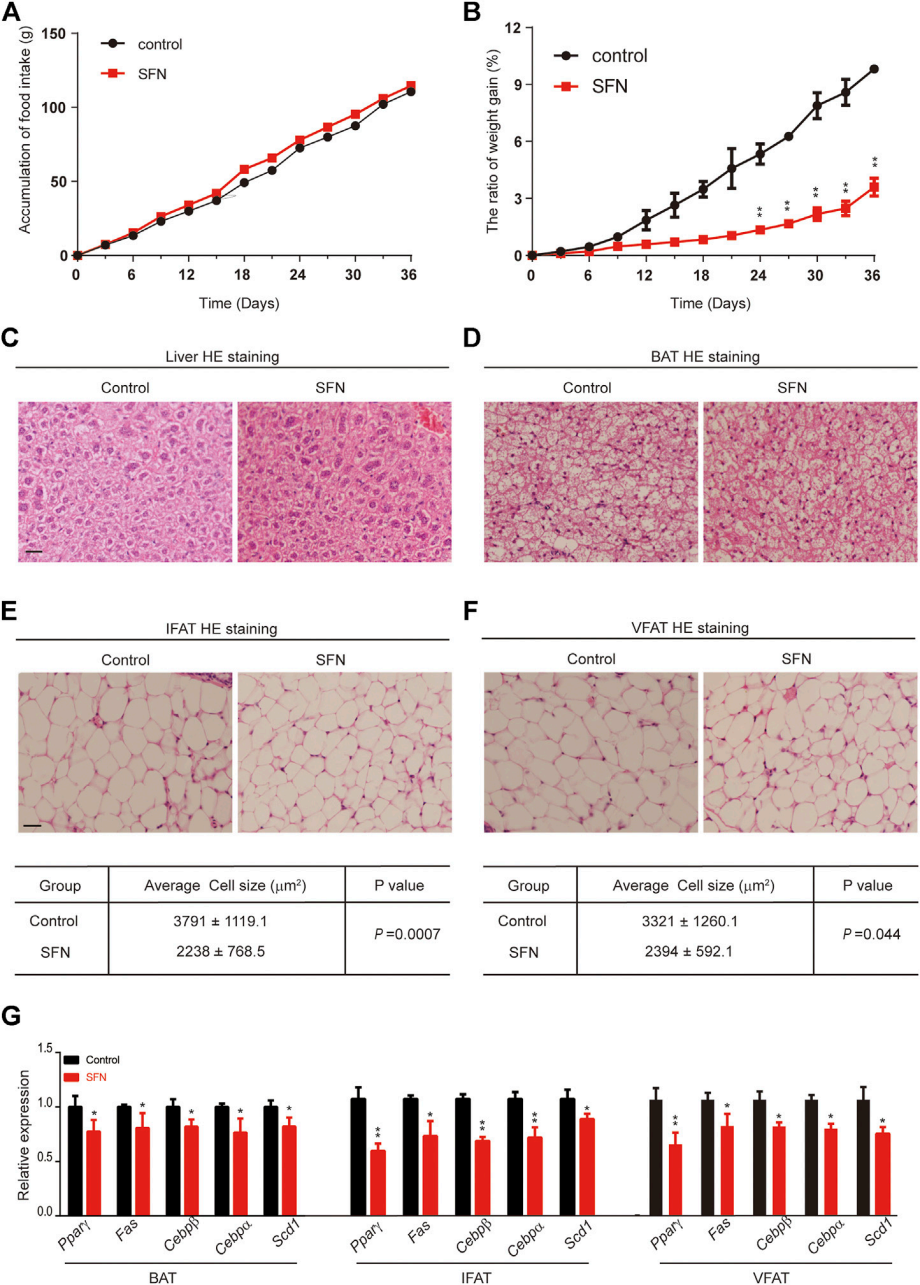
SFN impairs adipose cell differentiation in mice fed with normal diet. **(A,B)** SFN did not influence food intake but decreased weight gain. *n* = 7/group, **p < 0.01 vs. control. **(C,D)** Histology analysis of the effect of SFN on the cell morphology of the liver and BAT. **(E,F)** SFN decreased the adipose cell size of IWAT and VWAT. **(G)** SFN decreased the adipogenesis gene expression in WAT. Scar bar = 50 μm. All the results were expressed in graph with mean ± SD. Statistical significance was evaluated by Student t test. * and ** represent the significant difference at *p* < 0.05, *p* < 0.01 vs control group.

To explore the effect of SFN on weight gain in C57BL/6 mice, the 8-week-old female mice treated with 10 mg/kg/day SFN were noticeably protected from weight gain (Figure 3B). We assessed food intake in mice treated with SFN or vehicle. As shown in Figure 3A, SFN did not alter food intake. Therefore, the loss of weight gain is not the result of reduced calorie intake.

**FIGURE 4:**
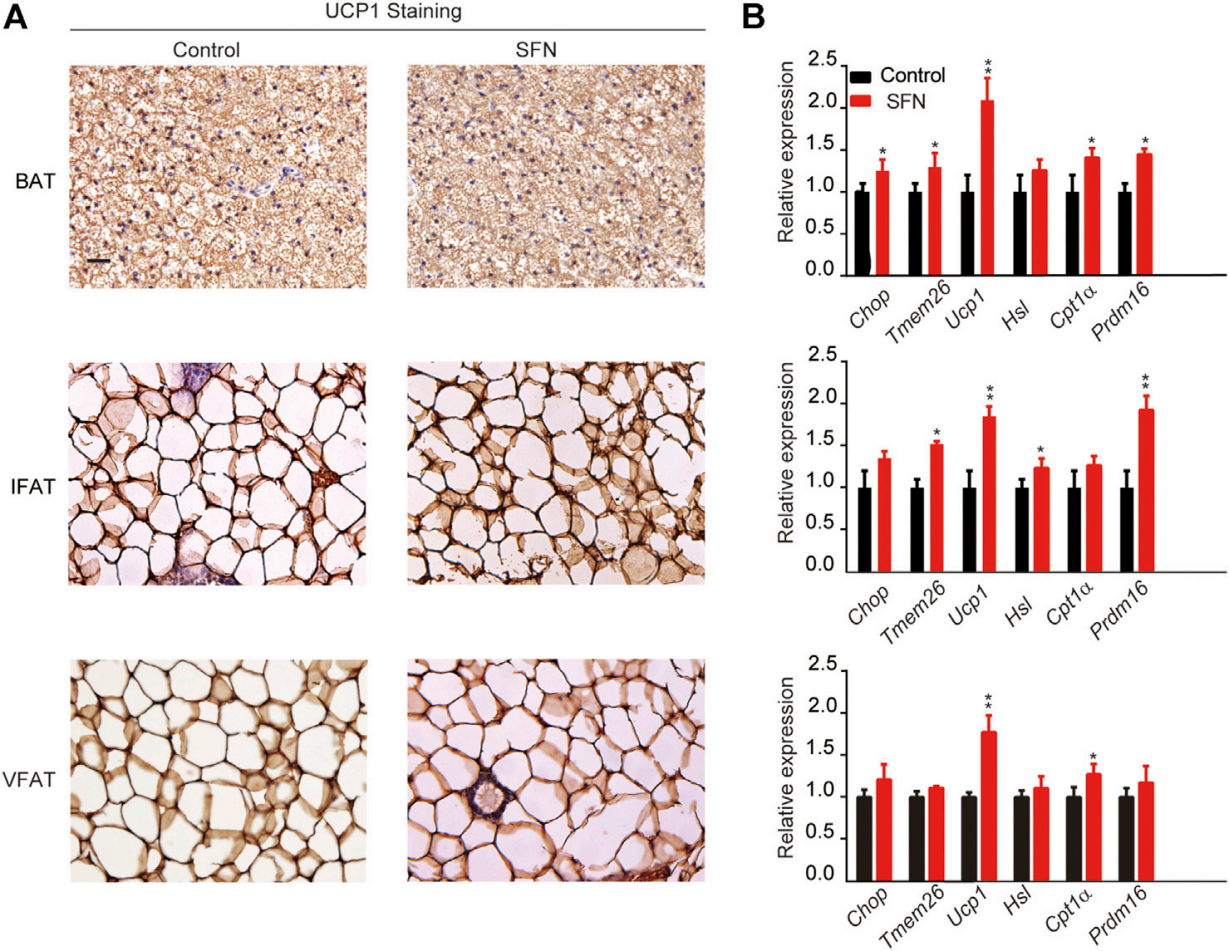
SFN promotes WAT browning in HFD-induced mice. **(A)** Immunostaining for UCP1 in the adipose tissue sections of BAT, IWAT, and VWAT from experiment mice. **(B)** SFN enhanced the browning gene expression in BAT, IWAT, and VWAT as determined by real-time qPCR assay. Scar bar = 50 μm. All the results were expressed in graph with mean ± SD. Statistical significance was evaluated by Student t test. * and ** represent the significant difference at *p* < 0.05, *p* < 0.01 vs control group.

Next, we performed HE staining on the sections of liver tissue, BAT, inguinal fat adipose tissue (IFAT), and visceral fat adipose tissue (VFAT). SFN had no toxic effect on body metabolism as the staining morphological analysis revealed no difference in liver tissue and BAT (Figures 3C,D). The average of adipocyte area in IFAT and VFAT in the SFN-treated group were considerably smaller than those in the vehicle-treated group (Figures 3E,F). The relative expression of adipogenic-specific genes was examined by qPCR (Figure 3G). SFN remarkably downregulated the mRNA expression of adipogenic-specific genes compared with vehicle control. Next, we examined the effect of SFN on the browning of adipose tissue. The immunohistochemistry staining showed that, in the SFN-treated group, the level of UCP1 in fat tissues was higher than in the vehicle-treated group (Figure 4A). The expression of browning marker genes indicated that brown fat marker genes (*Chop, Temem26, Ucp1, Pgc-1*α*, Prdm16, Hsl* and *Cpl1*α) were remarkably upregulated in fat tissues in the SFN group (Figure 4B).

### SFN-Treated Mice Are Protected Against HFD-Induced Obesity

**FIGURE 5:**
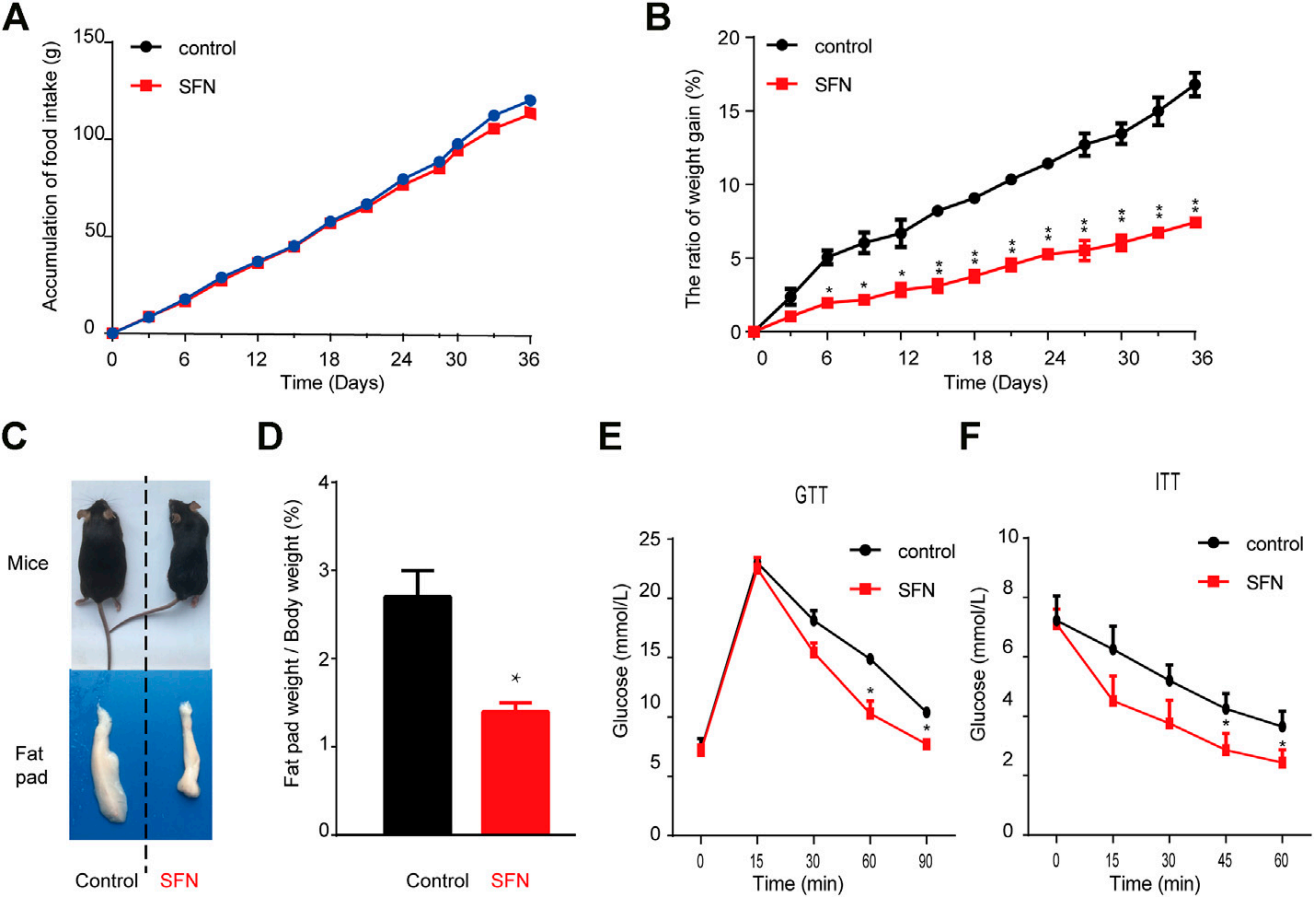
SFN prevented HFD-induced obesity. **(A,B)** SFN had no effect on food intake but decreased body weight gain, *n* = 7/group, *p < 0.05, **p < 0.01 vs. control. **(C)** Representative photograph of HFD-induced mice and SFN-treated HFD-induced mice **(D)** Percent of fat pad weight to the whole body weight. **(E,F)** GTT and ITT in control and SFN-treated groups. *n* = 7/group. All the results were expressed in graph with mean ± SD. Statistical significance was evaluated by Student t test. * and ** represent the significant difference at *p* < 0.05, *p* < 0.01 vs control group.

Next, we explored the effect of SFN on HFD-induced obesity in C57BL/6 mice. SFN (10 mg/kg/day) was intraperitoneally injected into female HFD fed C57BL/6 mice. After 36 days of injection, the SFN- and vehicle-treated groups had no difference in total food intake (Figure 5A). However, the rate of body weight gain in the SFN-treated group was considerably lower than that of the vehicle-treated group (Figure 5B). In addition, SFN substantially decreased the volume and weight of inguinal fat compared with the vehicle-treated group (Figures 5C,D). GTT and ITT were performed to investigate the effect of SFN on metabolism. The SFN-treated mice showed remarkable improvement in glucose and insulin tolerance compared with vehicle control (Figures 5E,F).

**FIGURE 6:**
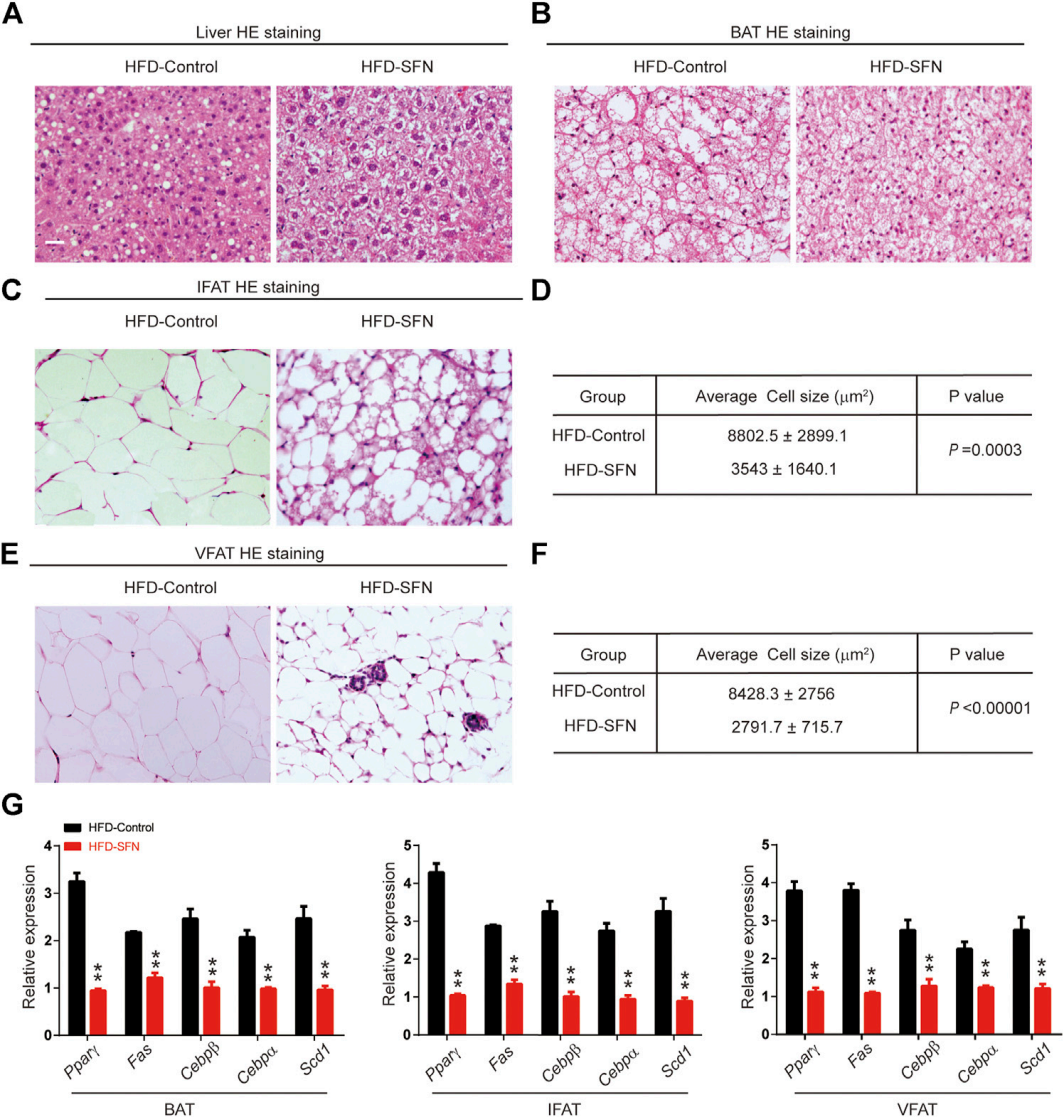
SFN prevented lipid accumulation in HFD-induced mice. **(A,B)** HE staining showed the effect of SFN on the liver and BAT. **(C–F)** SFN reduced the lipid droplets and the size of adipose cell from IWAT and VWAT. **(G)** Changes in the expression adipogenesis genes in different adipose tissues from HFD-induced obese mice without SFN treatment. Scar bar = 50 μm. All the results were expressed in graph with mean ± SD. Statistical significance was evaluated by Student t test. * and ** represent the significant difference at *p* < 0.05, *p* < 0.01 vs control group.

HE staining showed that the morphology of liver cells was affected by HFD (Figure 6A). The steatosis in livers caused by HFD was largely ameliorated after SFN treatment. HE staining was also performed to detect the morphological alteration in fat tissues. The results showed that the BAT cells of the SFN-treated group had more multilocular lipid droplets (Figure 6B), and that the cell sizes of IFAT (Figures 6C,D) and VFAT (Figures 6E,F) remarkably decreased in the SFN group. Aside from the effects on morphological characteristics, the mRNA level of adipogenesis-specific genes was substantially reduced in the SFN group (Figure 6G). Thus, we drew the conclusion that SFN could inhibit adipogenesis in HFD-fed mice.

### SFN Triggers WAT Browning in Mice to Prevent HFD-Induced Obesity

**FIGURE 7:**
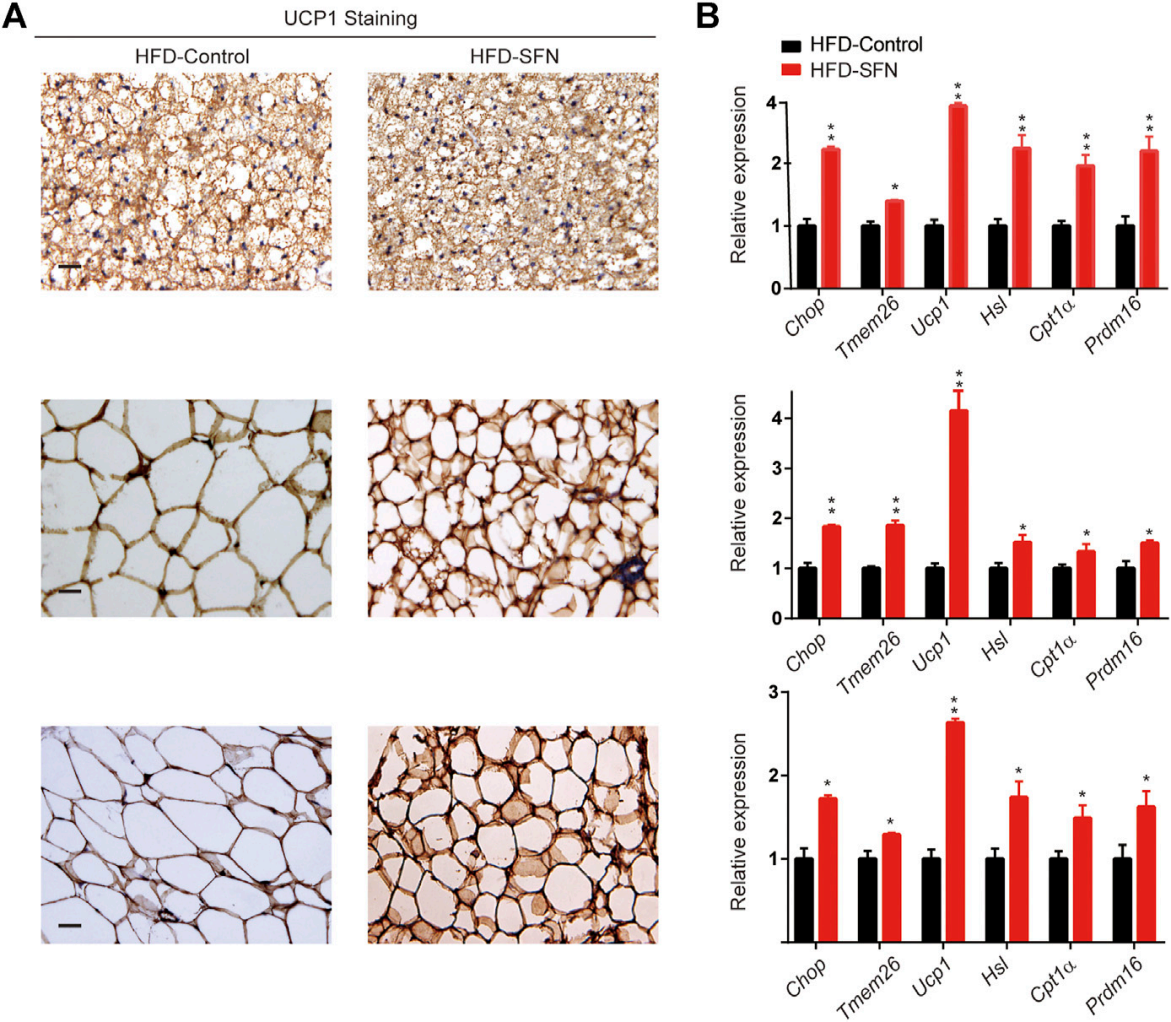
SFN induced WAT browning in HFD-induced mice. **(A)** Immunostaining for UCP1 in adipose tissue sections of BAT, IWAT, and VWAT from experiment mice. **(B)** SFN enhanced the expression of browning genes in BAT, IWAT, and VWAT as determined by real-time qPCR assay. Scar bar = 50 μm. All the results were expressed in graph with mean ± SD. Statistical significance was evaluated by Student t test. * and ** represent the significant difference at *p* < 0.05, *p* < 0.001 vs control group.

Immunohistochemistry was performed to understand the mechanism by which SFN exerted its fat-browning effect. SFN treatment upregulated the expression of UCP1 in adipose tissues (Figure 7A). Furthermore, we analyzed the expression of brown adipocyte marker genes that exhibited altered expression levels in adipose tissues (Figure 7B). The expression of brown adipocyte marker genes were substantially elevated compared with the control group.

The browning effect of SFN is associated with the regulation of the NRF2/PGC-1α and MAPK pathways and the increase in mitochondrial biogenesis.

**FIGURE 8:**
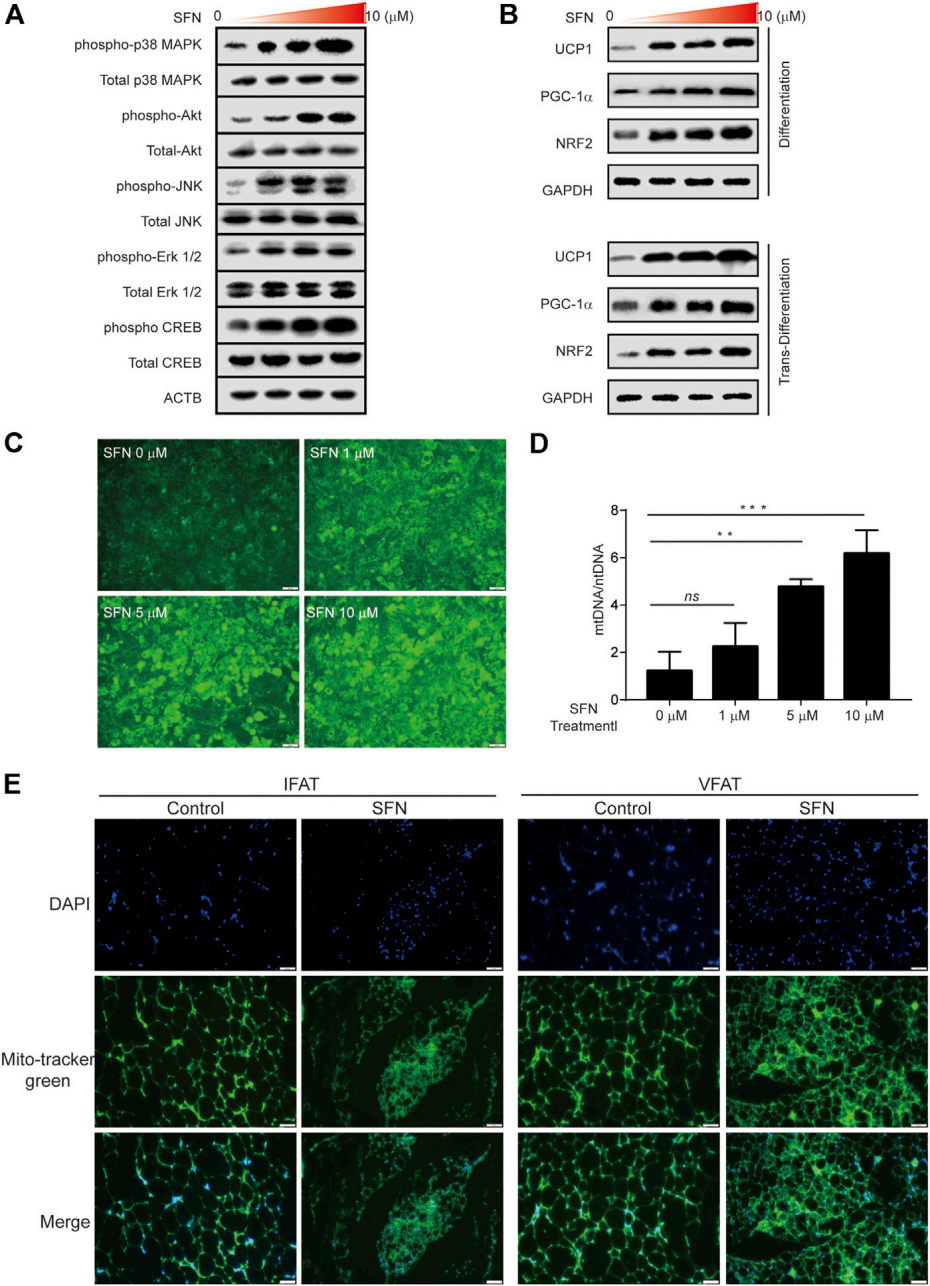
SFN promoted the browning of adipocytes through mitochondrial biogenesis. **(A)** SFN activated the MAPK and PKA–CREB pathway. **(B)** SFN promoted the expression of UCP1, PGC-1α, and NRF2 in the adipocyte differentiation and trans-differentiation periods. **(C)** Distribution of adipocyte mitochondria as assessed by MitoTracker Green staining. **(D)** The copy number of mtDNA per adipocyte was assessed by real-time qPCR. **(E)**. Scar bar = 50 μm. The content of adipocyte mitochondria assessed by MitoTracker Green staining in the adipose tissue section of HFD-induced mice. All the results were expressed in graph with mean ± SD. Statistical significance was evaluated by ANOVA test. *, ** and *** represent the significant difference at *p* < 0.05, *p* < 0.001 and *p* < 0.0001. *ns* represent not significant.

C3H10T1/2 cells were treated with SFN for 1 h to further verify the function of SFN on WAT browning. The results showed that the MAPK pathway was activated during browning (Figure 8A). Then, we investigated the protein level of the Nrf2/PGC-1α pathway in C3H10T1/2 cells treated with SFN in adipogenic differentiation and trans-differentiation. As shown in Figure 8B, SFN markedly increased the levels of these proteins in a dose-dependent manner. (The original films were shown in Supplementary Material). The results demonstrated that the NRF2/PGC-1α and MAPK pathways could be activated by SFN. The MitoScene Green staining was used to observe mitochondrial biosynthesis in SFN-treated cells (Figure 8C). The results showed that fluorescence intensity was increased by SFN treatment, and the cells treated with 10 μM SFN showed the highest fluorescence intensity. The ratio of mtDNA to nuclear DNA was dramatically increased by SFN (Figure 8D). Furthermore, we found that the mitochondrial biosynthesis in the WAT of SFN-treated mice fed with HFD was elevated compared with that in vehicle control (Figure 8E) by immunostaining.

## Discussion

There are mainly three types of adipose tissue in mammals: WAT, BAT, and beige adipose tissue (Hildebrand et al., 2018). WAT and BAT play different roles in lipid metabolism (Srivastava and Veech, 2019). Beige adipose develops postnatally and is highly inducible. Beige adipocyte recruitment, like white-to-brown adipocyte, is mediated by multiple factors, such as sympathetic nervous system activation and pharmacological conditions (Paulo and Wang, 2019). Metabolic disease, including obesity, can be prevented by activating the activities of brown and beige adipocytes, which has received much more attention (Heeren and Scheja, 2018). In this study we examined the effects of SFN on adipogenic differentiation in C3H10T1/2 preadipocytes and lipid metabolism regulation in HFD-fed obese C57BL/6 mice. We identified that SFN effectively inhibits the preadipocytes’ differentiation and enhances the mature adipocytes’ trans-differentiation into beige cells. Moreover, our *in vivo* experiment results confirmed that SFN treatment substantially decreased the adipocyte size and body weight gain, and further prevented HFD-induced obesity through the browning of adipocytes *via* mitochondrial biogenesis and the activation of *Ucp1* and *Pgc1-*α.

The common way to prevent obesity is to inhibit adipogenesis (Roman et al., 2015). Here, we report that SFN showed a distinct anti-adipogenesis activity in adipocytes. SFN treatment can substantially inhibit the expression of adipogenesis-specific genes, such as *Ppar*γ*, Fas, Cebp*β and *Scd1*, *in vivo* and *in vitro* and reduce lipid accumulation in pre-adipocyte differentiation. In the present study, SFN drastically decreased adipocyte size in inguinal and visceral adipose tissues compared with the control in mice fed with normal diet or HFD; thus, SFN could inhibit adipogenesis and lipid accumulation remarkably.

Beige adipocytes are interspersed in WAT (Giralt and Villarroya, 2013). Recently, it was well known that adipocytes can trans-differentiate into beige adipocytes through browning (Bartelt and Heeren, 2014). Adipocyte browning can promote the energy metabolism and insulin resistance contributing to the body weight loss. (Carey and Kingwell, 2013). In an *in vivo* study, the fusion of lipid droplet can be enhanced by the inducer in differentiate medium, while the transformation of beige adipocyte is promoted by SFN treatment (Zhang et al., 2016). Similarly, we observed that SFN induced browning in HFD-induced obese mice. The inhibition of lipogenesis and the reduction of intracellular lipid content by SFN was resulting from the enhancement of energy expenditure *via* promoting the energy metabolism of adipocyte. We detected the beige adipocyte-related biomarkers to prove the formation of beige adipocyte formation after SFN treatment. In this study, SFN drastically increased the transcriptional and translational level of beige adipocyte-related genes including *Ucp1*, *Pgc1-*α and *Prdm16* compared with the control. These data revealed that SFN can suppress the formation of white adipocytes while stimulate the beige adipocyte formation.

No previous study has reported that SFN has anti-obesity activity in animal *via* adipocyte browning. In our research, we observed no changes in food intake accumulation, but body weight gain was attenuated in the SFN-treated group. The expression of *Ppar*γ and *Cebp*α were induced by HFD in murine adipose tissue. Accordingly, the inhibition of these two genes in adipose tissue can prevent obesity (Choi et al., 2014). By regulating various genes responsible for adipogenesis *Ppar*γ and *Cebp*α play essential roles in adipocyte metabolism (Wang et al., 2020). In parallel with these previous studies, our data indicated that the expression of *Ppar*γ and *Cebp*α can be drastically inhibited by SFN in inguinal and visceral adipose tissues and even in BAT. We observed similar effect of SFN in both female and male mice while white fat browning is more pronounced in female mice (data not shown). Gender differences may be involved in the white fat browning process (Chang et al., 2018; Shao et al., 2020) and potential mechanism needs more and further research.

UCP1 is abundant in the mitochondria of BAT, which plays a crucial role in the production and consumption of energy as well as beige fat (Ruan, 2020). The browning of white adipose, where WAT trans-differentiate into beige adipose tissue, is directly associated with increasing the quantity of mitochondria and promoting mitochondrial function in adipose tissue (M. Liu et al., 2019). Here, we showed that SFN has a positive effect on mitochondrial contents and induces the elevation of the gene expression levels of related mitochondrial biogenesis biomarkers, including *Pgc1-*α. The activation of these biomarkers could furthermore contribute to the expression of downstream transcription factors. We used immunofluorescence by MitoTracker Green to assess mitochondrial content and better understand the importance of SFN in mitochondrial biogenesis. SFN-treated adipocytes showed remarkably increased mitochondrial activity. Adipocytes treated with SFN appeared to have a remarkable improvement in mitochondrial activity. In general, these findings indicated that the differentiation of adipocyte had a direct relation with the adjustment of the energy metabolism. In addition, SFN enhance the browning of adipocyte *via* upregulating *Ucp1* expression.

Oxidative stress is considered to play a vital role in cell injury and metabolic disorders in different kinds of diseases (Vona et al., 2019). It is believed that mitochondria are major production place of reactive oxygen species (ROS) (Lejri et al., 2019). Lipid overaccumulation results in a significant activation of intracellular ROS accumulation, leading to intensified oxidative process in obesity. This alteration can damage mitochondrial function, causing higher reactive oxygen species production (Brunetta et al., 2020). SFN has been proven to be an effective compound that prevents oxidative stress through the activation of *Nrf2* (D. Li et al., 2020). *Pgc-1*α and *Nrf2* were two of the important modulating factors in mitochondrial biogenesis (Kasai et al., 2020). Moreover, it has been reported that SFN could improve mitochondrial content, which means SFN could alter mitochondrial biogenesis (Lei et al., 2019). Correspondingly, SFN exhibited an adverse effect against the decline of mtDNA content in mice fed with HFD. In the present study, the inhibition of adipogenesis and the promotion of adipocyte browning by SFN was related to its antioxidant capacity for inducing mitochondrial biogenesis.

Due to the cellular expansion, the MAPK pathway is downregulated in the early adipogenesis and would be suppressend for adipogenesis. (Jang et al., 2015). However, in our study, at the period of trans-differentiation, the MAPK pathway was upregulated because of SFN treatment. Some studies confirmed the positive role of MAPK pathway in browning of adipocytes in 3T3-L1 cells. (Bae and Kim, 2020). Our present study also supported that the augmentation of browning by SFN treatment is involved with the activation of MAPK pathway at the late period of adipogenesis. Hence, MAPK pathway possibly has a beneficial effect on the promotion of browning adipogenesis.

## Conclusion

In the present study, we demonstrated that SFN could attenuate adipogenesis and explored the underlying mechanisms. SFN triggered the browning of C3H10T1/2 adipocytes *via* upregulating the expression of biomarkers concerned with brown adipocyte as well as mitochondrial biogenesis *in vivo* and *in vitro,* and inhibiting mitochondrial oxidative stress. Our study suggests that SFN, as a nutritional factor, is a promising medicine in the battle against obesity and various metabolic disorders *via* promoting the browning of white fat and improving glucose metabolism. Thus SFN may be used in new strategies for the prevention and inhibition of obesity and related diseases.

## Data Availability

The raw data supporting the conclusions of this article will be made available by the authors, without undue reservation, to any qualified researcher.
