# Supplementary material for: The Protective Effects of Sulforaphane on High-Fat Diet-Induced Obesity in Mice Through Browning of White Fat
Source: Front Pharmacol. 2021 Apr 29;12:665894. doi: 10.3389/fphar.2021.665894 (PMC8116735; doi:10.3389/fphar.2021.665894)
Supplement: Supplementary file 1 [file DataSheet1.docx]

Supplementary Figures:


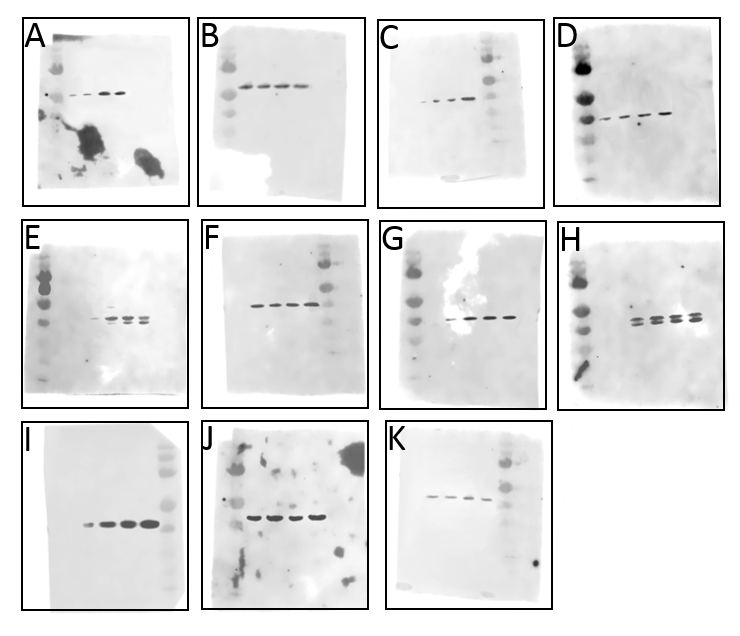


**Figure S1.** SFN activated the MAPK and PKA–CREB pathway. The full blots of C3H10T1/2 cells treated with SFN at the concentration of 0 μM, 1 μM, 5 μM, 10 μM respectively. (A) phos-Akt(Cell Signaling Technology; 4060); (B) Akt(Cell Signaling Technology; 4685); (C) phos-p38(Cell Signaling Technology; 4511); (D) p38(Cell Signaling Technology; 8690); (E) phos-JNK(Cell Signaling Technology; 4668); (F) JNK(Cell Signaling Technology; 9252); (G) phos-Erk(Cell Signaling Technology; 4370); (H) Erk(Cell Signaling Technology; 4695); (I) phos-CREB(Cell Signaling Technology; 9198); (J) CREB(Cell Signaling Technology; 9197); (K) ACTB( 20536-1-AP; proteintech). All the primary antibodies were incubated at the concentration of 1:1000. The images were presented using ECL luminous fluid (Advansta, America) on the imaging system (Li-cor Odyssey, America). PageRuler™ Prestained Protein Ladder, 10 to 180 kDa (26616)


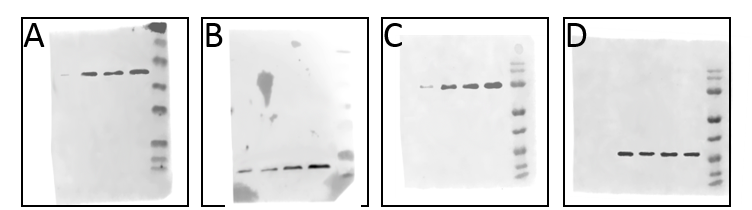


**Figure S2.** SFN promoted the expression of UCP1, PGC-1α, and NRF2 in the adipocyte differentiation. The full blots of C3H10T1/2 cells treated with SFN at the concentration of 0 μM, 1 μM, 5 μM, 10 μM respectively. (A) UCP-1 (Ab10983; Abcam); (B) PGC1α (66369-1-Ig; proteintech); (C) NRF2 (16396-1-AP; proteintech); (D) GAPDH (10494-1-AP; proteintech). All the primary antibodies were incubated at the concentration of 1:1000. The images were presented using ECL luminous fluid (Advansta, America) on the imaging system (Li-cor Odyssey, America). PageRuler™ Prestained Protein Ladder, 10 to 180 kDa (26616)


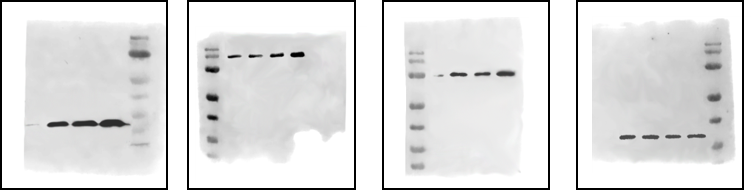


**Figure S3.** SFN promoted the expression of UCP1, PGC-1α, and NRF2 in the adipocyte trans-differentiation. The full blots of C3H10T1/2 cells treated with SFN at the concentration of 0 μM, 1 μM, 5 μM, 10 μM respectively. (A) UCP-1(Ab10983; Abcam); (B) PGC1α (66369-1-Ig; proteintech); (C) NRF2 (16396-1-AP; proteintech); (D) GAPDH (10494-1-AP; proteintech). All the primary antibodies were incubated at the concentration of 1:1000. The images were presented using ECL luminous fluid (Advansta, America) on the imaging system (Li-cor Odyssey, America). PageRuler™ Prestained Protein Ladder, 10 to 180 kDa (26616)
